# *In Vivo* Kinetics and Biotransformation of Aflatoxin B_1_ in Dairy Cows Based on the Establishment of a Reliable UHPLC-MS/MS Method

**DOI:** 10.3389/fchem.2021.809480

**Published:** 2021-12-24

**Authors:** Wenbo Guo, Zhichen Fan, Kai Fan, Jiajia Meng, Dongxia Nie, Emmanuel K. Tangni, Zenghe Li, Zhihui Zhao, Zheng Han

**Affiliations:** ^1^ School of Chemistry, Beijing University of Chemical Technology, Beijing, China; ^2^ Institute for Agro-food Standards and Testing Technology, Shanghai Key Laboratory of Protected Horticultural Technology, Shanghai Academy of Agricultural Sciences, Shanghai, China; ^3^ Organic Contaminants and Additives, Chemical and Physical Health Risks, Sciensano, Leuvensesteenweg, Brussels, Belgium

**Keywords:** aflatoxins, *in vivo* kinetics, biotransformation, dairy cow, UHPLC-MS/MS

## Abstract

The *in vivo* kinetics of aflatoxin B_1_ (AFB_1_) and its carry-over as aflatoxin M_1_ (AFM_1_) in milk as well as the toxin loads in the tissue of dairy cows were assessed through a repetitive feeding trial of an AFB_1_-contaminated diet of 4 μg kg^−1^ body weight (b.w.) for 13 days. This was followed by a clearance period that ended with a single dose trial of an AFB_1_-contaminated diet of 40 μg kg^−1^ b.w. An ultra-high performance liquid chromatography tandem mass spectrometry method was developed and successfully validated by the determination of linearity (*R* ^2^ ≥ 0.990), sensitivity (lower limit of quantification, 0.1–0.2 ng ml^−1^), recovery (79.5–111.2%), and precision relative standard deviation (RSD) ≤14.7%) in plasma, milk, and various tissues. The repetitive ingestion of AFB_1_ indicated that the biotransformation of AFB_1_ to AFM_1_ occurred within 48 h, and the clearance period of AFM_1_ in milk was not more than 2 days. The carry-over rate of AFM_1_ in milk during the continuous ingestion experiment was in the range of 1.15–2.30% at a steady state. The *in vivo* kinetic results indicated that AFB_1_ reached a maximum concentration of 3.8 ± 0.9 ng ml^−1^ within 35.0 ± 10.2 min and was slowly eliminated from the plasma, with a half-life time (T_1/2_) of 931.1 ± 30.8 min. Meanwhile, AFM_1_ reached a plateau in plasma (0.5 ± 0.1 ng ml^−1^) at 4 h after the ingestion. AFB_1_ was found in the heart, spleen, lungs, and kidneys at concentrations of 1.6 ± 0.3, 4.1 ± 1.2, 3.3 ± 0.9 and 5.6 ± 1.4 μg kg^−1^, respectively. AFM_1_ was observed in the spleen and kidneys at concentrations of only 0.7 ± 0.2 and 0.8 ± 0.1 μg kg^−1^, respectively. In conclusion, the *in vivo* kinetics and biotransformation of AFB_1_ in dairy cows were determined using the developed UHPLC-MS/MS method, and the present findings could be helpful in assessing the health risks to consumers.

## Introduction

Aflatoxin B_1_ (AFB_1_), primarily produced by *Aspergillus flavus*, *Aspergillus parasiticus*, and *Aspergillus nomius*, is frequently found in different feeds and their raw materials (Kumar et al., 2016; Frazzoli et al., 2017). AFB_1_ has been classified as a group Ι. Carcinogen by the International Agency for Research on Cancer (IARC) (Global Health, 2012) because of its hepatic, carcinogenic, teratogenic, mutagenic, immunosuppressive, and reproductive toxicities to livestock and poultry (Gross-Steinmeyer and Eaton, 2012; Iqbal et al., 2019). Aflatoxin M_1_ (AFM_1_), derived from the 4-hydroxylated metabolite of AFB_1_ (chemical structures shown in Supplementary Figure S1), is also a potential human carcinogen classified as group Ι by IARC (Ostry et al., 2017), and it is usually secreted into milk after the ingestion of AFB_1_-contaminated diets.

In recent decades, there have been many reports on the natural occurrence of AFB_1_ in feeds and AFM_1_ in milk and milk products (Natour et al., 1991; Han et al., 2013; Canestrari et al., 2016). The amount of AFM_1_ in milk and AFB_1_ in feeds consumed by animals could lead to health risks to consumers (Gonçalves et al., 2017). Therefore, the maximum AFB_1_ limits have been set as 5 μg kg^−1^ for compound feeds and 20 μg kg^−1^ for all feed materials in EU (European Commission (EC), 2003), 20 μg kg^−1^ for different feeds in the United States (Food and Agriculture Organization (FAO), 2004), and 10 μg kg^−1^ in concentrate supplementary feeds and 50 μg kg^−1^ in feed materials in China (State Administration for Market Regulation, 2017). For AFM_1_, China, several other Asian countries, and the United States have set a maximum level of 0.5 μg kg^−1^ in raw milk and dairy products (Food and Drug Adminstration (FDA), 1996; ASEAN, 2015; National Health Commission of the people’s Republic of China, 2017), while a considerably lower level (0.05 μg kg^−1^) is stipulated in the EU (European Commission (EC), 2006).

Considering the widespread occurrence and intense toxicity, the *in vivo* kinetics of AFB_1_ have been attracting more increasing attention. A few of studies have demonstrated that dietary AFB_1_ is rapidly absorbed into the gastrointestinal tract of different animals and partially transformed to AFM_1_ in milk for ruminant animals, such as cows and sheep, which are the primary source of AFM_1_ in milk (Battacone et al., 2003; Zaghini et al., 2005; Corcuera et al., 2012). The rate of dietary AFB_1_ carry-over as AFM_1_ in milk ranged from 0.3 to 6.2% for cows (Applebaum et al., 1982; Frobish et al., 1986) and from 1.3 to 2.9% for sheep (Battacone et al., 2005; Battacone et al., 2009). Similarly, the *in vivo* toxicokinetics of AFB_1_ have also been studied in different model animals, including rats, mice, and monkeys (Wong and Hsieh, 1980; Bastaki et al., 2010; Corcuera et al., 2012) but not in dairy cow. To date, there is no literature on the distribution of AFB_1_ in different tissues and organs of dairy cows, which poses potential health risks to consumers. Notably, distinct differences in previous reports about the carry-over rate and *in vivo* kinetics of AFB_1_ in various animal species were due to differences in AFB_1_-delivery types, metabolic pathways, and animal susceptibility. Moreover, outdated detection methods, such as thin-layer chromatography (Stubblefield, 1986) and enzyme-linked immunosorbent assay (Diaz et al., 2004) have occasionally resulted in discrepant and contradictory results in earlier studies owing to complex sample pretreatment, lower sensitivity, and incomplete methodology.

The main objective of this study was to develop and validate an accurate and sensitive ultra-high performance liquid chromatography tandem mass spectrometry (UHPLC-MS/MS) method to analyze the *in vivo* kinetics and biotransformation of AFB_1_ in AFB_1_-contaminated diet. Based on the model of dairy cows, the results will contribute to the understanding of the effects of dietary AFB_1_ loads on its carry-over in milk, such as AFM_1_, as well as distribution, and elimination of AFB_1_ *in vivo*. The illustration of the kinetics and biotransformation of AFB_1_ is shown in Supplementary Figure S2.

## Materials and Methods

### Chemicals and Reagents

Methanol, acetone, and acetonitrile (all HPLC grade) were purchased from Merck (Darmstadt, Germany). Ammonium acetate (HPLC grade) was obtained from Sigma-Aldrich (St. Louis, MO, United States). Water was filtered using a Millipore system (Millipore, Billerica, MA, United States). AFB_1_ (2.03 μg ml^−1^), and AFM_1_ (0.5 μg ml^−1^) of analytical standard were purchased from Romer Labs (Union, MO, United States).

### Preparation of Contaminated Diets

To produce AFB_1_-contaminated maize, an AFB_1_-producing strain (*Aspergillus flavus* 01) was isolated and identified at the mycotoxin research laboratory of Shanghai Academy of Agricultural Sciences, followed by cultivation on maize grains at 28°C for 28 days. The maize culture was then sterilized at 121°C, dried at 40°C for 60 h, and ground into powder. The concentrations of AFB_1_ in contaminated maize flour and total mixed rations (TMR) feed were accurately determined according to the previously developed UHPLC-MS/MS method (Guo et al., 2017). Subsequently, 8.3 and 83 g of the obtained maize culture containing 240 mg kg^−1^ AFB_1_ were blended with 200 g of AFB_1_-free TMR feed to develop two AFB_1_ contaminated diets: Diet A, 4 μg kg^−1^ body weight (b.w.) and Diet B, 40 μg kg^−1^ b.w. for the animal trials, respectively. After finishing the diets, the animals were fed AFB_1_-free TMR feed. The control group was directly fed AFB_1_-free TMR feed.

### Animals and Diet Management

Five Holstein dairy lactating cows (b.w. = 500 ± 10 kg, 30–32 weeks of calving) were purchased from Zhangxueping Dairy Farm (Nanjing, China). Before the experiments began, the dairy cows were given feed and water daily for a week for acclimatization. The dairy cows were randomly divided into an experimental group (three cows) and a control group (two cow). TMR feed (20 kg) per cow per day was administered in equal doses at 0,700 h and 1700 h according to the methods of the National Research Council to ensure milk production of ≥10 kg. The feed was divided into small portions and given to the cows several times to ensure that all feed was consumed. The health of all the dairy cows was monitored continuously during the experimental period. This experiment was approved by the Animal Ethics Committee of Shanghai Academy of Agricultural Science (Shanghai, China) (SYXK (Hu) 2015-0007) and Zhuozhou Jierong Bio-Technology Co., Ltd. (Zhuozhou City, Hebei, China) (SYXK (Ji) 2018-003).

### Experiment Design and Sample Collection

During the carry-over trial, dairy cows were repeatedly fed with AFB_1_- contaminated TMR feed (4 μg kg^−1^ b.w.) or AFB_1_-free TMR feed for 13 days. All the dairy cows were milked at 0,730 h and 1730 h, and the milk yield was recorded. Milk samples (10 ml) were collected twice daily according to the volume of daily milk production. All milk samples were stored at −20°C until analysis. After a 30-days clearance period, a higher single dose of AFB_1_ in contaminated TMR feed (40 μg kg^−1^ b. w.) was administered to the experimental cows. Successive milk samples (10 ml) were collected at 0.5, 1, 3, 6, 9, 24, 36, 48, 72, and 96 h after the administration of AFB_1_-contaminated diet for further carry-over analysis of AFB_1_. Simultaneously, 5 ml of blood from each cow was drawn from the caudal vein at 10, 35, 45, 60, 120, 180, 240, 360, 540, 720, 1,440, 2,160, and 2,880 min for the *in vivo* kinetic study of AFB_1_. Each blood sample was immediately collected in a heparinized tube and centrifuged at 2,739×g for 15 min. Subsequently, aliquots of plasma were transferred into clean tubes and stored at −20°C until use. After another 30-days clearance period, all the cows were sacrificed 6 h after the oral administration of AFB_1_ (40 μg kg^−1^ b.w.). Tissue samples from cows, including heart, liver, spleen, lung, and kidney, were collected and stored in liquid nitrogen until analysis. Blank milk, blood, and tissue samples from the control group were collected to establish the analytical method.

### UHPLC-MS/MS Analysis

After thawing at room temperature, 200 µL of milk, plasma, and tissue homogenates, which were homogenized with normal saline (1/3, m/v), were separately transferred into a 2.5-ml centrifuge tube. Acetone (1.4 ml) of was added for protein precipitation and target extraction. The mixtures were blended by vortexing for 30 s and centrifuged at 16,099 ×g for 5 min. Subsequently, 1 ml of the supernatant was evaporated under a soft stream of nitrogen gas at 40°C, and the residues were re-dissolved in 200 µL of acetonitrile/water containing 5 mmol L^−1^ ammonium acetate (20/80, v/v). The residues were then filtered through a 0.22 μm membrane filter for UHPLC-MS/MS analysis.

UHPLC-MS/MS analysis was performed on a Waters ACQUITY UPLC system coupled with an AB SCIEX Triple Quad TM 5500 mass spectrometer. LC separation was achieved on a Poroshell EC_18_ column (2.1 × 100 mm, 2.7 μm, Agilent, United States) with methanol (A) and 5 mmol L^−1^ ammonium acetate (B) as the mobile phase. The flow rate was 0.4 ml min^−1^ and a total of 8 min of gradient elution procedure was applied as follows: initial 10% A; 0.5 min, 10% A; 1.5 min, 50% A; 5.0 min, 90% A; 6.0 min, 90% A; 6.2 min, 10% A; and 8.0 min, 10% A. The injection volume was 3 μL, and the column temperature was 40°C.

Electrospray ionization was used in positive (ESI^+^) mode with the following parameters: ion spray voltage, 5500 V; source temperature, 500°C; ion source gas 1 (GS1), 50 psi; ion source gas 2 (GS2), 50 psi; and collision gas (CAD), 8 psi. The multiple reaction monitoring (MRM) mode was used for the quantification and confirmation of AFB_1_ and AFM_1_ with the parameters listed in Supplementary Table S1.

### Carry-Over Analysis

The carry-over rate of AFB_1_ to AFM_1_ was calculated according to the following formula:

$$Carry­over\;rate\;\left(\%\right)=\frac{m_{milk}\;\times \;C_{AFM1}}{m_{TMR}\;\times \;C_{AFB1}}\times 100\%$$

The m_milk_ and m_TMR_ are the milk yield (kg) and quantity of AFB_1_- contaminated TMR feed (kg) daily, respectively. C_AFM1_ and C_AFB1_ are the concentrations of AFM_1_ in milk (µg kg^−1^) and AFB_1_ in the diet (µg kg^−1^), respectively.

The graphs of concentration–time curves were prepared using Origin 9.0, (La Jolla, CA, United States), which were then used to illustrate the carryover of AFB_1_ and AFM_1_ in milk. All data are presented as mean ± standard deviation (SD).

### *In Vivo* Kinetics and Tissue Distribution

After oral administration, *in vivo* kinetics of AFB_1_ was performed with DAS 2.0 (Shanghai, China) using non-compartmental analysis. AUC_(0-t)_ and AUC_(0-∞)_ are the areas under the plasma concentration–time curve from time 0–2,160 min and infinity, respectively. MRT_(0-t)_ and MRT_(0-∞)_ are the mean residence times from time 0–2,160 min and infinity, respectively, where T_1/2_ is the terminal elimination half-life. C_0_ and C_max_ are the initial and maximal plasma concentrations, respectively. T_max_ is the time to maximal plasma concentration. All data are presented as mean ± SD.

The concentrations of AFB_1_ and AFM_1_ in different tissues from individual dairy cows, including the heart, liver, spleen, lungs, and kidneys were determined.

### Method Validation

The analytical method for detecting AFB_1_ and AFM_1_ in plasma, milk, and various tissues, such as the heart, liver, spleen, lungs, and kidneys, was validated according to the guidelines on bioanalytical method validation provided by the European Medicines Agency (Blume et al., 2011). Linearity was evaluated in neat solvent and in plasma, milk, and various tissues spiked with AFB_1_ and AFM_1_ at concentrations of 0.1–200 ng ml^−1^. The calibration curves were drawn by plotting responses *versus* analyte concentrations, and the acceptable criterion of R^2^ was ≥0.99. The lower limit of quantification (LLOQ) was the lowest concentration point of the calibration curves, which is typically defined as a theoretical signal-to-noise (S/N) ratio of 10. The lower limit of detection (LLOD) was the lowest concentration that could be determined and defined as a theoretical S/N ration of 3. Note that S/N=SD/*k*, where SD is the standard deviation of the blank (*n* = 6) and *k* is the slope of the matrix-matched calibration curve. The recovery and precision were evaluated in blank samples spiked with LLOQ, low, intermediate, and high levels (LLOQ, 1, 50, and 200 ng ml^−1^ for plasma and milk; LLOQ, 1, 50, and 200 μg kg^−1^ for various tissues, respectively) of AFB_1_ and AFM_1_ in six replicates. RSD values on the same day and on five successive days were used to evaluate the intra- and inter-day precision, respectively. The short-term (room temperature for 8 h) and long-term (-20°C for 20 days) stability of spiked plasma and tissue samples (1 and 50 ng ml^−1^ for plasma and milk, 1 and 50 μg kg^−1^ for various tissues), as well as the stability after three freeze–thaw cycles, were evaluated to ensure that the concentrations of AFB_1_ and AFM_1_ were not affected. In addition, blank, spiked, and real plasma, milk, and spleen collected after AFB_1_ oral administration were individually analyzed and evaluated for specificity.

## Results and Discussion

### Optimization of Extraction Solvent

In the current study, three different solvents (methanol, acetonitrile and acetone) at different extraction volumes (0.6, 1.0, 1.4, and 1.8 ml, respectively) were compared for the spiked milk samples (50 ng ml^−1^). The extraction efficiency was evaluated according to the following formula:

Extraction efficiency = extraction recovery × matrix effect × 100%

As shown in Supplementary Table S2, the highest extraction efficiency of 77.5 and 89.4% was achieved for AFB_1_ and AFM_1_, respectively, when 1.4 ml of acetone was selected. Similar trends were observed for AFB_1_ and AFM_1_ in plasma and different tissue samples. Therefore, 1.4 ml of acetone was selected as the extraction solvent for protein precipitation and target extraction.

### Method Validation

**FIGURE 1:**
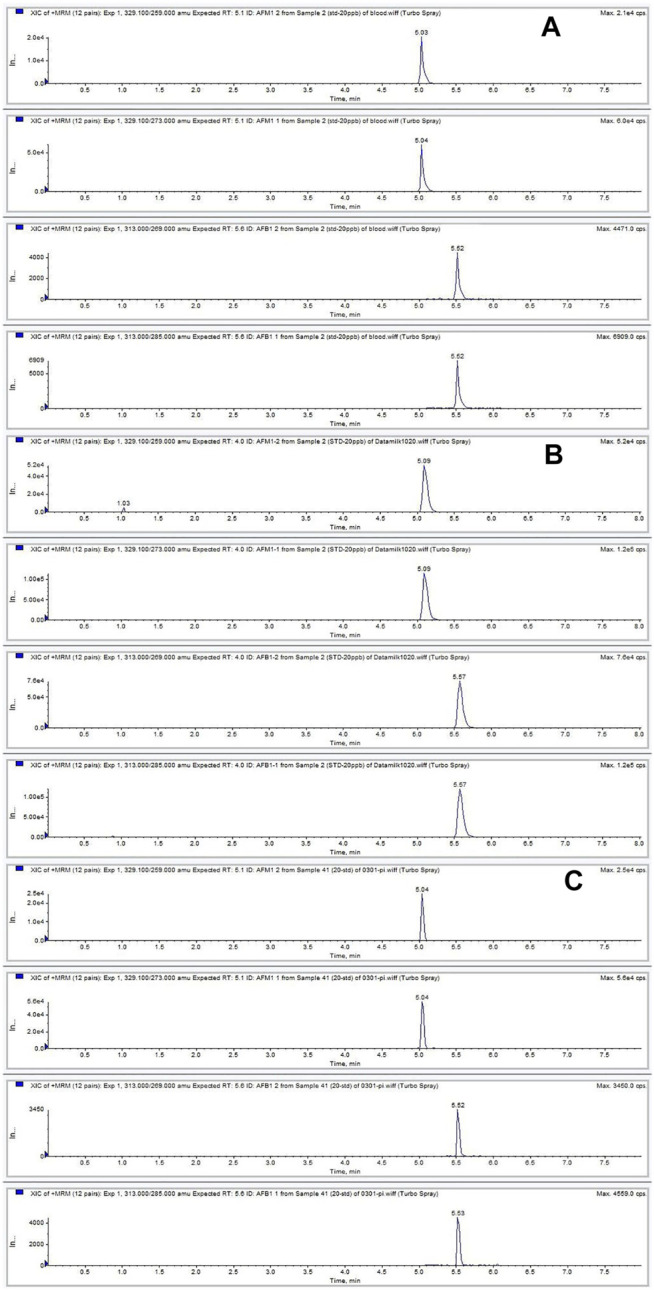
Chromatograms of AFB_1_ and AFM_1_ detected in plasma **(A)**, milk **(B)**, and spleen **(C)** contaminated at 20 ng ml^−1^. The retention times of AFB_1_ and AFM_1_ were 5.0 and 5.5 min, respectively.

Good linear relationships were obtained with correlation coefficients (*R* ^2^) > 0.99 in neat solvent and blank plasma, milk, and tissues (Supplementary Table S3). The LLODs and LLOQs for AFB_1_ and AFM_1_ in different matrices were in the range of 0.03–0.2 ng ml^−1^ (μg kg^−1^) and 0.1–0.5 ng ml^−1^ (μg kg^−1^), respectively. Satisfactory recoveries and precisions for AFB_1_ and AFM_1_ at various spiking levels are listed in Table 1. The recoveries ranged from 79.5 to 102.3% for milk; 82.8–107.9% for plasma; 88.7–111.2% for heart; 83.3–109.6% for liver; 86.8–104.8% for spleen; 85.8–103.0% for lung; and 85.1–106.6% for kidney. The intra- and inter-day RSDs were in the range of 3.0–12.3% and 6.8–14.7%, respectively, for various matrices, indicating the acceptable reproducibility of the proposed method. The concentration at each spiking level of all samples after the short-term, long-term, and three freeze–thaw cycle stability tests were in the range of 82.2–102.0% (Supplementary Table S4), which indicated that AFB_1_ and AFM_1_ in all the biological matrices were stable. Moreover, no endogenous interference was observed at the respective retention times of AFB_1_ (5.0 min) and AFM_1_ (5.5 min) in plasma, milk, and spleen matrices (Figure 1), verifying the good selectivity of this method.

**TABLE 1:** Recovery and intra- and inter-day precision of AFB_1_ and AFM_1_ in milk, plasma, and different tissues (*n* = 6).

Sample matrix	Aflatoxin	Spiking level (ng mL^−1^/μg kg^−1^)	Recovery (mean ± SD, %)	Intra-day precision (RSD, %)	Inter-day precision (RSD, %)
Milk	AFB_1_	LLOQ	85.3 ± 7.5	8.8	9.8
		1	79.5 ± 8.7	10.9	11.4
		50	86.4 ± 8.1	9.3	9.5
		200	98.7 ± 9.2	9.2	10.6
	AFM_1_	LLOQ	102.3 ± 10.4	10.1	12.5
		1	79.8 ± 8.4	10.5	11.3
		50	88.4 ± 7.8	8.8	9.7
		200	87.5 ± 6.5	7.4	10.3
Plasma	AFB_1_	LLOQ	90.7 ± 8.5	9.4	12.4
		1	82.8 ± 10.2	12.3	14.7
		50	92.4 ± 8.8	9.5	11.3
		200	94.1 ± 9.9	10.5	11.6
	AFM_1_	LLOQ	107.9 ± 11.2	10.3	14.0
		1	94.3 ± 3.4	3.6	9.7
		50	95.3 ± 5.8	6.1	10.2
		200	96.8 ± 2.9	3.0	13.6
Heart	AFB_1_	LLOQ	102.5 ± 4.1	4.0	6.8
		1	88.7 ± 4.5	5.1	8.1
		50	103.5 ± 3.9	3.7	9.9
		200	92.1 ± 8.7	9.5	10.3
	AFM_1_	LLOQ	111.2 ± 5.0	4.5	6.9
		1	99.2 ± 10.2	10.2	9.4
		50	96.3 ± 5.3	5.5	9.1
		200	94.5 ± 9.0	9.5	9.0
Liver	AFB_1_	LLOQ	104.1 ± 8.4	8.0	8.9
		1	93.0 ± 11.1	11.9	10.8
		50	93.3 ± 4.4	4.7	8.3
		200	98.3 ± 8.8	9.1	7.7
	AFM_1_	LLOQ	109.6 ± 9.2	7.4	8.3
		1	83.3 ± 5.5	6.6	10.2
		50	91.1 ± 11.1	12.1	11.2
		200	89.0 ± 7.0	7.9	8.4
Spleen	AFB_1_	LLOQ	104.8 ± 9.1	8.9	8.6
		1	86.8 ± 10.1	11.6	9.4
		50	100.0 ± 11.0	11.0	8.9
		200	94.5 ± 7.7	8.1	9.3
	AFM_1_	LLOQ	99.9 ± 8.6	8.6	9.3
		1	87.3 ± 7.3	8.4	9.3
		50	95.2 ± 11.6	12.1	9.9
		200	87.6 ± 11.3	12.9	11.2
Lung	AFB_1_	LLOQ	102.4 ± 11.9	11.6	12.4
		1	93.6 ± 10.8	11.6	12.1
		50	94.5 ± 7.2	7.6	9.7
		200	85.8 ± 7.2	8.4	8.7
	AFM_1_	LLOQ	103.0 ± 9.4	9.1	10.1
		1	89.4 ± 8.4	9.3	9.5
		50	97.5 ± 6.6	6.7	8.4
		200	95.5 ± 6.9	7.2	8.0
Kidney	AFB_1_	LLOQ	100.9 ± 10.1	10.0	11.2
		1	85.1 ± 9.7	11.3	11.7
		50	94.9 ± 8.0	8.4	9.8
		200	91.4 ± 6.6	7.2	8.3
	AFM_1_	LLOQ	106.6 ± 6.1	5.7	8.2
		1	87.0 ± 6.4	7.3	11.2
		50	91.6 ± 5.9	6.5	8.5
		200	92.2 ± 9.2	10.0	10.8

### Carry-Over Rate of AFB_1_ to AFM_1_ in Milk

**FIGURE 2:**
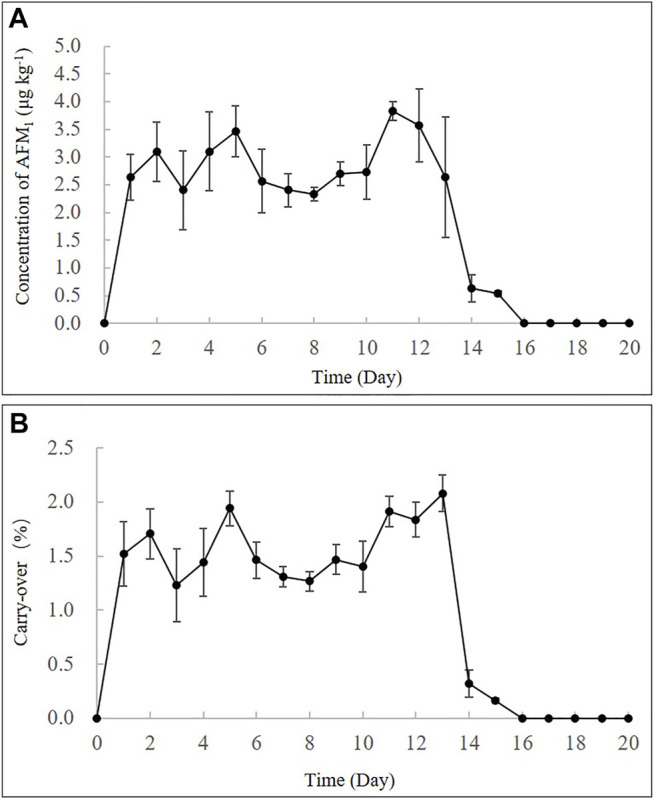
Time-concentration profiles **(A)** and carry-over rate % **(B)** of AFM_1_ in milk after continuous ingestion of AFB_1_ (4 μg kg^−1^ b.w.) for 13 days.

The repetitive ingestion of 4 μg kg^−1^ b.w. of AFB_1_ for 13 days (intoxication period) demonstrated that the concentrations of AFM_1_ in the milk increased rapidly from the first day, with concentrations remaining in the range of 2.6–3.8 μg kg^−1^ till day 13 (Figure 2A). As presented in Supplementary Table S5, this result was similar to that previously reported in cows that were fed a diet containing ∼ 86 μg AFB_1_ daily for 7 days (Britzi et al., 2013). After the intoxication period (13 days), the cows were fed AFB_1_-free feeds, and the milk was collected for 7 days (clearance period). The concentration of AFM_1_ in milk decreased gradually and could not be detected after 2 days. These results corresponded with those of previous studies that reported the clearance period typically lasted less than 3 days for AFB_1_ (Diaz et al., 2004). As depicted in Figure 2B, the carry-over rate of AFM_1_ in milk during the continuous ingestion experiment was in the range of 1.15–2.30% at a steady state, which was consistent with the range of 1–3% that has been reported in previous studies (Diaz et al., 2004; Van Eijkeren et al., 2006; Masoero et al., 2007) (Supplementary Table S5).

**FIGURE 3:**
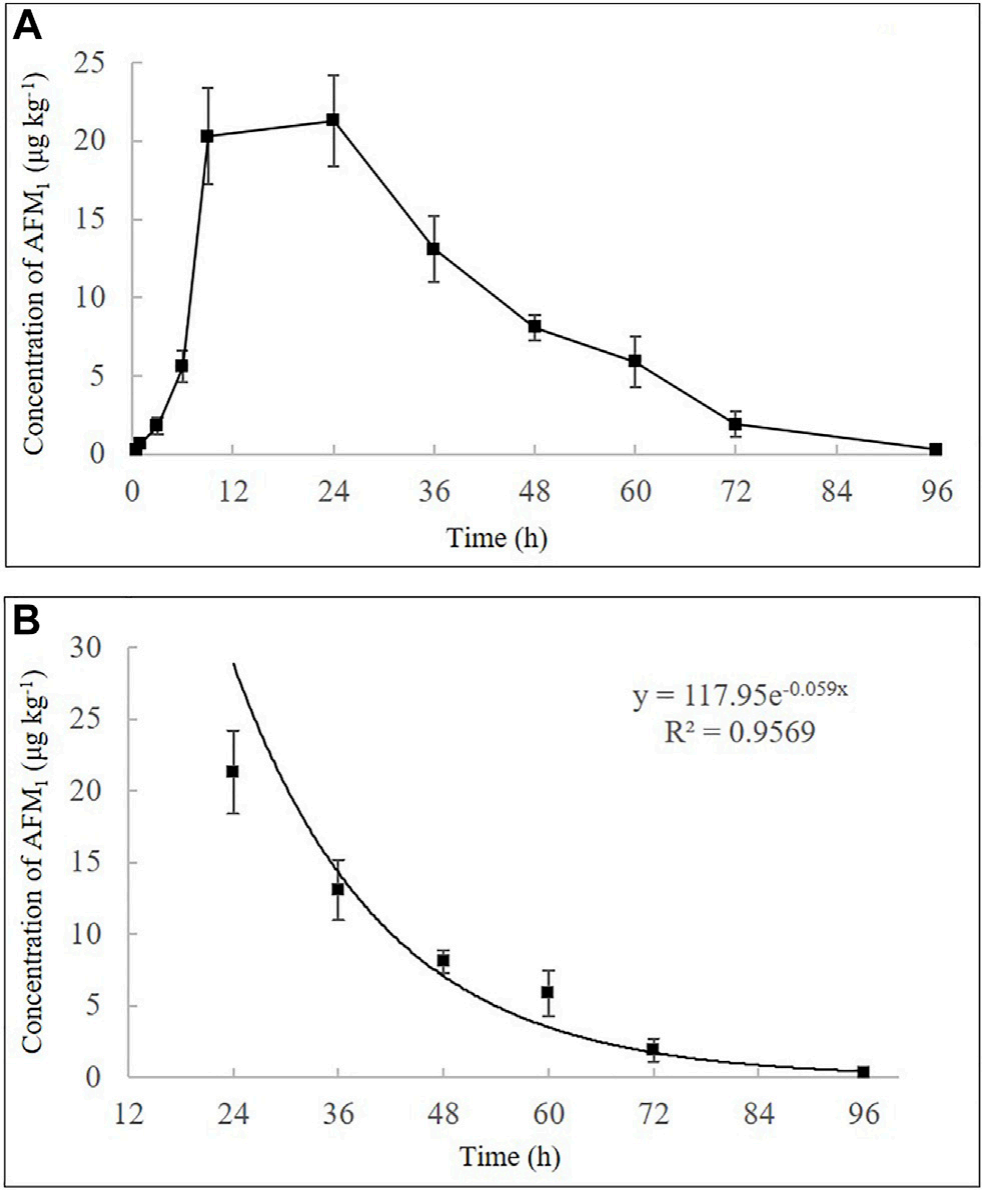
Time-concentration profiles of AFM_1_ in milk after a single oral administration of AFB_1_ (40 μg kg^−1^ b.w.) **(A)** and the disappearance pattern of AFM_1_ in the milk **(B)**. Values are presented as mean ± standard deviation, *n* = 3.

Furthermore, a high single dose (40 μg kg^−1^ b.w.) of feed artificially contaminated with AFB_1_ showed that AFM_1_ in milk increased rapidly and the highest concentration of AFM_1_ was observed at 24 h (21.3 ± 2.9 μg kg^−1^) (Figure 3A). After its plateau, AFM_1_ concentration decreased rapidly and could not be detected after 96 h. The disappearance pattern of AFM_1_ in milk is depicted in Figure 3B, and the disappearance of AFM_1_ in milk can be expressed as: $y\;=\;117.95e^{-0.059x},\;R^{2}\;=\;0.9569$ . Overall, no significant differences were observed in the carry-over of AFB_1_ to AFM_1_ with different administration approaches and concentrations, similar to the results of previous studies on cows and sheep. However, the observed plateaus and clearance periods of AFM_1_ in milk were partially variable (Supplementary Table S5). These variations may be related to the different dietary sources of AFB_1_, for example, pure AFB_1_ or naturally AFB_1_-contaminated corn, cottonseed, and ground-peanut meal, varying levels of AFB_1_ dose, as well as the differences between individual animals (Battacone et al., 2003; Battacone et al., 2012; Sumantri et al., 2012).

### *In Vivo* Kinetics

**FIGURE 4:**
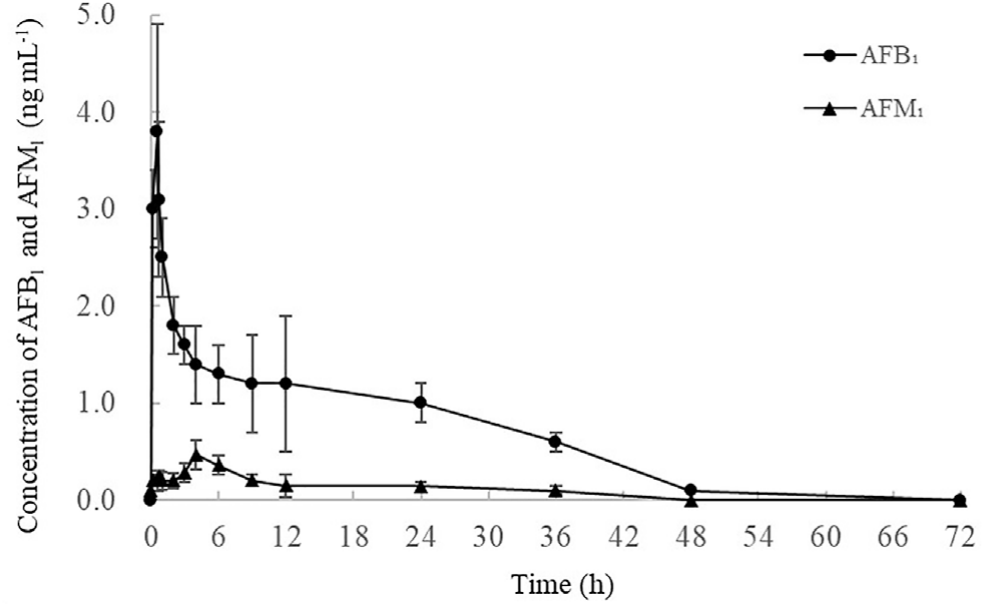
Time-concentration profile of AFB_1_ and AFM_1_ in the plasma after a single oral administration of AFB_1_ (40 μg kg^−1^ b.w.) (*n* = 3). Values are presented as mean ± standard deviation.

The concentration–time profiles of AFB_1_ and AFM_1_, as well as the toxicokinetic parameters in plasma after a single dose (40 μg kg^−1^ b.w.) of AFB_1_ are presented in Figure 4 and Table 2. The results indicated that AFB_1_ was rapidly absorbed in all studied cows with the highest concentrations (C_max_ = 3.8 ± 0.9 ng ml^−1^) approximately 35.0 ± 10.2 min after oral administration. Meanwhile, AFB_1_ was rapidly eliminated in cows (T_1/2_ = 931.1 ± 30.8 min) and transformed into AFM_1_, which plateaued in the plasma (0.5 ± 0.1 ng ml^−1^) at 4 h after ingestion. As presented in Supplementary Table S5, the values of the primary kinetic parameters in this study were significantly different from those of other animals, such as rats, mice, monkeys, and broiler chickens (Bastaki et al., 2010; Cui et al., 2017). This can be attributed to many factors, including the differences in AFB_1_ intake, gastrointestinal absorption, animal health, and particularly in the activity of cytochrome P450 (CYP450) enzymes, which play an important role in the transformation of AFB_1_ to AFM_1_ in the liver (Applebaum et al., 1982; Gross-Steinmeyer and Eaton, 2012).

**TABLE 2:** Primary toxicokinetic parameters of AFB_1_ after a single oral administration (40 μg kg^−1^ b.w.) to dairy cows (*n* = 3).

Toxicokinetic parameter^a^	Unit	Mean ± SD
AUC (0–t)	ng min mL^−1^·	1763.3 ± 132.5
AUC (0–∞)	ng min mL^−1^	2,162.7 ± 359.6
MRT (0–t)	min	703.5 ± 56.6
MRT (0–∞)	min	1,220.7 ± 94.1
T_1/2_	min	931.1 ± 30.8
C_0_	ng mL^−1^	0
C_max_	ng mL^−1^	3.8 ± 0.9
T_max_	min	35.0 ± 10.2

a AUC_0-t_ = area under the plasma concentration-time curve from time 0–2,160 min, AUC_0-∞_ = area under the plasma concentration-time curve from time 0 to infinity, MRT (0–t) = mean residence time from time 0–2,160 min, MRT (0–∞) = mean residence time from time 0 to infinity; T_1/2_ = terminal elimination half-life; C_0_ = plasma concentration at time 0; C_max_ = maximal plasma concentration; T_max_ = time to maximal plasma concentration; SD, standard deviation.

### Tissue Distribution

After a single oral dose of AFB_1_ (40 μg kg^−1^ b.w.), all tissues were analyzed *via* the validated UHPLC-MS/MS method. The concentrations of AFB_1_ in the heart, spleen, lungs and kidneys were 1.6 ± 0.3, 4.1 ± 1.2, 3.3 ± 0.9 and 5.6 ± 1.4 μg kg^−1^, respectively. Although the liver is typically considered the most susceptible organ for AFB_1_, neither aflatoxin was detected in all the live samples. It is likely that AFB_1_ in the liver was completely cleared because of the time taken between last feed and sacrifice (Corcuera et al., 2012; Cui et al., 2017). Moreover, AFM_1_ was observed in the spleen and kidneys at concentrations of 0.7 ± 0.2 and 0.8 ± 0.1 μg kg^−1^, respectively. In summary, these results verified the effects of AFB_1_ and AFM_1_ accumulation in different tissues, particularly in the spleen and kidneys, which could pose health risks for both dairy cows and consumers.

## Conclusion

An accurate and reliable UHPLC-MS/MS method was established and validated for the simultaneous determination of AFB_1_ and AFM_1_ in the plasma, milk, and tissues of dairy cows. And the method was applied to investigate *in vivo* kinetics and biotransformation of AFB_1_ in dairy cows. A rapid absorption, distribution, and excretion of AFB_1_ was observed in dietary cows with relatively high residues detected in kidneys, lungs, heart, and spleen. A certain amount of AFB_1_ (1.15–2.30%) could also be transformed to AFM_1_, as another important risk factors and then excreted into milk. This comprehensive study will be of great value in the evaluation and control of AFB_1_ contamination in feeds to reduce the health risks posed to both humans and animals.

## Data Availability

The original contributions presented in the study are included in the article/Supplementary Material, further inquiries can be directed to the corresponding authors.
