# Supplementary material for: In Vivo Kinetics and Biotransformation of Aflatoxin B1 in Dairy Cows Based on the Establishment of a Reliable UHPLC-MS/MS Method
Source: Front Chem. 2021 Dec 24;9:809480. doi: 10.3389/fchem.2021.809480 (PMC8740645; doi:10.3389/fchem.2021.809480)
Supplement: Supplementary file 1 [file DataSheet1.docx]

***In vivo* kinetics and biotransformation of aflatoxin B_1_ in dairy cows based on the establishment of a reliable UHPLC-MS/MS method**

Wenbo Guo^1,2^, Zhichen Fan^2^, Kai Fan^2^, Jiajia Meng^2^, Dongxia Nie^2^, Emmanuel K. Tangni^3^, Zenghe Li^1,*^, Zhihui Zhao^2^, Zheng Han^2,*^

^1^ School of Chemistry, Beijing University of Chemical Technology, No. 15 North Third Ring East Road, 100028, Beijing, China

^2^ Institute for Agro-food Standards and Testing Technology, Shanghai Key Laboratory of Protected Horticultural Technology, Shanghai Academy of Agricultural Sciences, No. 1000 Jinqi Road, 201403, Shanghai, China

^3^ Organic Contaminants and Additives, Chemical and Physical Health Risks, Sciensano, Leuvensesteenweg 17, 3080 Tervuren, Belgium

* To whom correspondence should be addressed.

E-mail address: lizh@mail.buct.edu.cn; hanzheng@saas.sh.cn.

**Figure S1** Chemical structures of AFB_1_ and AFM_1_

**
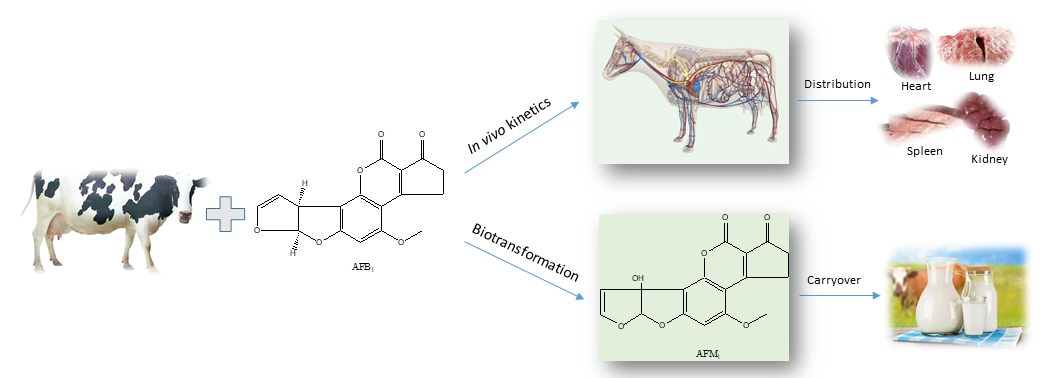
**

**Figure S2** The illustration of the kinetics and biotransformation of AFB_1_

**Table S1** MS/MS parameters for AFB_1_ and AFM_1_

**Table S2** The extraction efficiency of various extraction solvents in milk

**Table S3** Linearity and sensitivity of AFB_1_ and AFM_1_ in milk, plasma, and different tissues.

**Table S4** Stability of AFB_1_ and AFM_1_ in milk, plasma, and different tissues (n=6)

**Table S5** Comparison between previously reported studies of kinetics and biotransformation of AFB_1_.

**Table S1** MS/MS parameters for AFB_1_ and AFM_1_

| Mycotoxin | RT  (min) | Precursor ion  (*m/z*) | Product ion  (*m/z*) | DP  (V) | EP  (V) | CE  (V) | CXP  (V) |
| --- | --- | --- | --- | --- | --- | --- | --- |
| AFB_1_ | 5.6 | 313.0 | 285.0*/269.0 | 84 | 10 | 30/43 | 8 |
| AFM_1_ | 4.3 | 329.1 | 273.0*/259.0 | 100 | 15 | 31/30 | 12 |

* Quantitative ion； RT, retention time; DP, declustering potential; EP, entrance potential; CE, collision energy; CXP, collision cell exit potential

**Table S2** The extraction efficiency of various extraction solvents in milk

| Extraction solvent | Volume (μL) | extraction efficiency (%) | |
| --- | --- | --- | --- |
|  |  | AFB_1_ | AFM_1_ |
| Methanol | 0.6 | 45.6 | 49.5 |
|  | 1.0 | 65.4 | 66.7 |
|  | 1.4 | 73.8 | 82.6 |
|  | 1.8 | 70.1 | 76.7 |
| Acetonitrile | 0.6 | 35.6 | 45.1 |
|  | 1.0 | 63.9 | 67.9 |
|  | 1.4 | 69.8 | 78.6 |
|  | 1.8 | 65.3 | 71.4 |
| Acetone | 0.6 | 39.8 | 45.7 |
|  | 1.0 | 55.3 | 62.5 |
|  | 1.4 | 77.5 | 89.4 |
|  | 1.8 | 71.6 | 81.5 |

**Table S3** Linearity and sensitivity of AFB_1_ and AFM_1_ in milk, plasma, and different tissues.

| Matrix | Mycotoxin | Linear Range | Slope | Intercept | R^2^ | LLOD | LLOQ |
| --- | --- | --- | --- | --- | --- | --- | --- |
|  |  | (ng/mL) |  |  |  | （ng mL^-1^ /µg kg^-1^） | |
| Milk | AFB_1_ | 0.2-200 | 6787 | 655 | 0.993 | 0.08 | 0.2 |
|  | AFM_1_ | 0.1-200 | 8997 | -453 | 0.995 | 0.03 | 0.1 |
| Plasma | AFB_1_ | 0.2-200 | 8892 | -922 | 0.998 | 0.08 | 0.2 |
|  | AFM_1_ | 0.1-200 | 9881 | -732 | 0.996 | 0.03 | 0.1 |
| Heart | AFB_1_ | 0.2-200 | 1474 | 577 | 0.995 | 0.08 | 0.2 |
|  | AFM_1_ | 0.1-200 | 13701 | -226 | 0.997 | 0.03 | 0.1 |
| Liver | AFB_1_ | 0.2-200 | 911 | 1706 | 0.991 | 0.08 | 0.2 |
|  | AFM_1_ | 0.1-200 | 9870 | 554 | 0.997 | 0.03 | 0.1 |
| Spleen | AFB_1_ | 0.2-200 | 1261 | 1861 | 0.992 | 0.08 | 0.2 |
|  | AFM_1_ | 0.1-200 | 11869 | 560 | 0.998 | 0.03 | 0.1 |
| Lung | AFB_1_ | 0.2-200 | 1207 | 898 | 0.991 | 0.08 | 0.2 |
|  | AFM_1_ | 0.1-200 | 11439 | 90 | 0.999 | 0.03 | 0.1 |
| Kidney | AFB_1_ | 0.2-200 | 1073 | 1966 | 0.991 | 0.08 | 0.2 |
|  | AFM_1_ | 0.1-200 | 11049 | 526 | 0.999 | 0.03 | 0.1 |

**Table S4** Stability of AFB_1_ and AFM_1_ in milk, plasma, and different tissues (n=6)

| Matrix | Mycotoxin | Spiked level  (ng mL^-1^/µg kg^-1^) | Short-term stability ^a^ | Long-term stability ^b^ | Freeze-thaw stability ^c^ |
| --- | --- | --- | --- | --- | --- |
|  |  |  | (ng mL^-1^/µg kg^-1^) | | |
| Plasma | AFB_1_ | 1 | 82.8±10.2 | 90.8±10.6 | 89.0±8.8 |
|  |  | 50 | 92.4±8.8 | 94.2±9.2 | 90.6±12.2 |
|  | AFM_1_ | 1 | 94.3±3.4 | 80.9±7.9 | 89.2±2.8 |
|  |  | 50 | 95.3±5.8 | 99.9±7.1 | 82.2±12.9 |
| Heart | AFB_1_ | 1 | 85.2±9.9 | 90.1±8.7 | 87.8±8.2 |
|  |  | 50 | 100.3±9.1 | 85.1±3.6 | 91.9±2.7 |
|  | AFM_1_ | 1 | 87.7±9.2 | 90.9±7.2 | 85.9±8.0 |
|  |  | 50 | 96.4±7.6 | 97.6±7.3 | 95.8±9.7 |
| Liver | AFB_1_ | 1 | 86.7±10.2 | 92.5±9.9 | 91.6±7.5 |
|  |  | 50 | 100.2±8.1 | 89.4±9.3 | 98.7±10.8 |
|  | AFM_1_ | 1 | 88.3±13.2 | 86.2±7.4 | 93.3±8.2 |
|  |  | 50 | 94.3±7.7 | 95.3±8.8 | 94.2±6.6 |
| Spleen | AFB_1_ | 1 | 85.6±8.9 | 89.7±10.1 | 84.7±7.0 |
|  |  | 50 | 93.4±11.1 | 99.0±10.6 | 95.7±3.8 |
|  | AFM_1_ | 1 | 90.7±5.8 | 91.3±10.8 | 91.5±8.9 |
|  |  | 50 | 92.8±6.7 | 91.3±6.2 | 88.4±4.8 |
| Lung | AFB_1_ | 1 | 90.8±12.1 | 92.1±10.4 | 87.7±5.1 |
|  |  | 50 | 97.3±7.2 | 97.8±12.9 | 102.0±9.8 |
|  | AFM_1_ | 1 | 90.3±7.9 | 89.4±7.9 | 91.7±8.4 |
|  |  | 50 | 93.7±11.8 | 94.7±8.6 | 86.8±5.0 |
| Kidney | AFB_1_ | 1 | 87.7±10.1 | 87.2±8.3 | 91.1±6.9 |
|  |  | 50 | 89.5±9.0 | 90.1±11.2 | 96.1±5.9 |
|  | AFM_1_ | 1 | 92.4±11.6 | 95.9±7.1 | 86.3±5.1 |
|  |  | 50 | 98.7±11.9 | 90.5±9.6 | 94.9±9.0 |

^a^: stored at room temperature (RT) for 8 h; ^b^: stored at -20 °C for 20 days; ^c^: subjected to three freeze-thaw cycles.

**Table S5** Comparison between previously reported studies of kinetics and biotransformation of AFB_1_.

| Animal | Diet/dose/source | Carryover rate of AFM_1_ | C_max, AFM1_ | Clearance period | *In vivo* kinetics | | | | Tissue distribution | | | | | Refs. |
| --- | --- | --- | --- | --- | --- | --- | --- | --- | --- | --- | --- | --- | --- | --- |
|  |  |  |  |  | C_max, AFB1_ | T_1/2,_  _AFB1_ | T_1/2, AFM1_ | C_max, AFM1_ | lung | spleen | kidney | liver | heart |  |
| Israeli cows in late-lactation | ~86 μg AFB_1_ for 7 days, corn | 2.5% | 0.113μg/kg | 1 day | - | - | - | - | - | - | - | - | - | [31] |
| Israeli cows in mid-lactation |  | 5.8% | 0.075μg/kg |  |  |  |  |  |  |  |  |  |  |  |
| Lactation Holsteins | 55 ppb AFB_1_ for 5 days, corn | 2.25% | 1.24ppb | 3d | - | - | - | - | - | - | - | - | - | [32] |
| Lactating cow | 966 μg AFB_1_,  cottonseed | 1.38-2.33% | 0.62μg/L | - | - | - | - | - | - | - | - | - | - | [33] |
| Holstein cow (Various Milk yield ) | 98.10 ± 0.26 mg per cow per day, 10days, corn meal | 2.32, 2.70, 1.48 and 1.29% | 65.8, 61.9, 66.7 and 59.2 ng/L | 3d | - | - | - | - | - | - | - | - | - | [34] |
| Dairy  ewes | A single dose of pure AFB_1_ (2 mg) | 0.032% | - | - | - | - | - | - | - | - | - | - | - | [18] |
|  | 32, 64, and 128 μg for 14 d，pure AFB_1_ | 0.112% (average value) | 0.057, 0.226 and 0.331μg/kg |  |  |  |  |  |  |  |  |  |  |  |
| Goats | A single dose of 0.8 mg of pure AFB_1_ | 0.17% | - | 72 h | - | - | - | - | - | - | - | - | - | [35] |
| F344 rats | A single oral dose of 0.25 mg/kg bw AFB_1_ | - | - | - | 24.8 and 9.5 µg/L | - | - | - | - | - | Not detected | <LOQ | - | [20] |
| Broiler chickens | Dietary AFB_1_ (5.0 mg/kg feed) | - | - | - | - | - | - | - | - | - | 15 days in kidney | 11 days in liver | - | [38] |
| Pregnant mice | Single doses of pure AFB_1_ 20 mg/kg, intraperitoneally | - | - | - | 2.5 µg/mL | 15 min | 15 min | 2.2 µg/mL | - | - | - | - | - | [37] |
| Dairy cow in this study | Diet A, 4 μg kg^-1^ b.w. and Diet B, 40 μg kg^-1^ b.w.; maize | 1.15–2.30% at steady state | 3.8 and 21.3 μg kg^-1^ | 96 h | 3.8  ng mL^-1^ | 931.1 min | 240 min | 0.5 ng mL^-1^ | 3.3 μg kg^-1^ | 4.1 μg kg^-1^ | 5.6 μg kg^-1^ | - | 1.6 μg kg^-1^ | This study |
